# Enhanced neutrophil apoptosis accompanying myeloperoxidase release during hemodialysis

**DOI:** 10.1038/s41598-020-78742-z

**Published:** 2020-12-10

**Authors:** Taro Fukushi, Tae Yamamoto, Mai Yoshida, Emi Fujikura, Mariko Miyazaki, Masaaki Nakayama

**Affiliations:** 1 Tohoku University Hospital Division of Blood Purification, 1-1, Seiryo-cho, Aoba-ku, Sendai, Miyagi Prefecture Japan; 2 Graduate School of Medicine Division of Nephrology, Endocrinology and Vascular Medicine, Tohoku University, Sendai, Japan; 3 Research Division of Chronic Kidney Disease and Dialysis Treatment, Tohoku University Hospital, Sendai, Japan; 4 St Luke’s International Hospital, St Luke’s International University, Tokyo, Japan

**Keywords:** Biomarkers, Medical research, Nephrology, Pathogenesis

## Abstract

Biocompatibility of hemodialysis (HD) systems have been considerably improved. However, mortality and morbidity rates of patients have remained high, raising questions regarding the biocompatibility of current systems. In the present study, 70 patients on regular HD (51 males; mean age, 63 years; median duration of HD, 18 months) with high-performance membrane (polysulfone, 77%; polymethylmethacrylate, 23%) at Tohoku University Hospital were examined. Blood samples before and after HD, were subjected to measure apoptosis cells of white blood cells, plasma levels of the following molecules: myeloperoxidase (MPO), pentraxin 3 (PTX3), angiogenin, complements, and 17 cytokines. The main findings were as follows: significant decreases in leukocyte counts by dialysis, significant increases in apoptosis-positive leukocytes by dialysis (neutrophils and monocytes), and significant decrease in plasma angiogenin accompanying increase in plasma MPO and PTX3 levels, with no or only marginal changes in plasma pro-inflammatory cytokine levels and complement products by dialysis. The findings underlined the unsolved issue of bio-incompatibility of HD systems, and suggest the possible pathology of neutrophil apoptosis accompanying MPO release for the development of microinflammation in patients on HD.

## Introduction

Mortality risk among chronic dialysis patient is excessively high, with cardiovascular disease (CVD) and infection as the main causes of death^1^. Intrinsic and extrinsic pathological factors are thought to be involved in this elevated mortality rate; the former include underlying kidney disease, comorbidities, nutrition^2^, and control of the uremic state of the patient^3^, while the latter include the bio-compatibility of materials in the treatment system, such as the dialyzer membrane^4^, and dialysis water quality^5^.

Biocompatibility in this case refers to biological reactions induced between blood and the dialyzer or dialysate. These reactions primarily include complement activation, production of diverse cytokines such as interleukin (IL)-1β and tumor necrosis factor (TNF)-α by mononuclear leukocytes, and respiratory burst with degranulation and apoptosis of neutrophils. The cellulose membrane is one of the classic factors for bio-incompatibility, as the structure of cellulose contains hydrophilic hydroxyl groups, which strongly stimulate both activation of complement, and release of cytokines by mononuclear cells^6^. These reactions have been assumed to contribute to chronic inflammation and oxidative stress, leading to a higher risk of CVD and infectious disease in dialysis patients through injury to endothelial cells and immunocompetent cells^7^.

Various developments have been made toward improving dialysis biocompatibility, such as the use of improved cellulose or synthetic polymer membranes as dialyzer materials^8^, and purification of dialysis water to reduce endotoxin contamination^9^. However, the influence of HD on pro-inflammatory cytokines have been conflicting. There are studies which showed lack of increase in serum cytokine levels after HD sessions, indicating improved biocompatibility for mononuclear cells in current dialysis system^9–11^. While some studies showed increased serum cytokine levels by HD^12^, and enhanced cytokine transcription of peripheral mononuclear cells during HD^13^. In the real clinical aspect, e.g. recent trends in Japan, infectious morbidity in dialysis patients has not been suppressed, which raises questions regarding a possible injury to leukocytes by HD. However, data on biocompatibility by current HD system has been limited.

The purpose of this study was to examine the impact on peripheral leukocytes during hemodialysis (HD), with particular focus on neutrophils in patients using synthetic polymer membranes.

## Results

**Figure 1:**
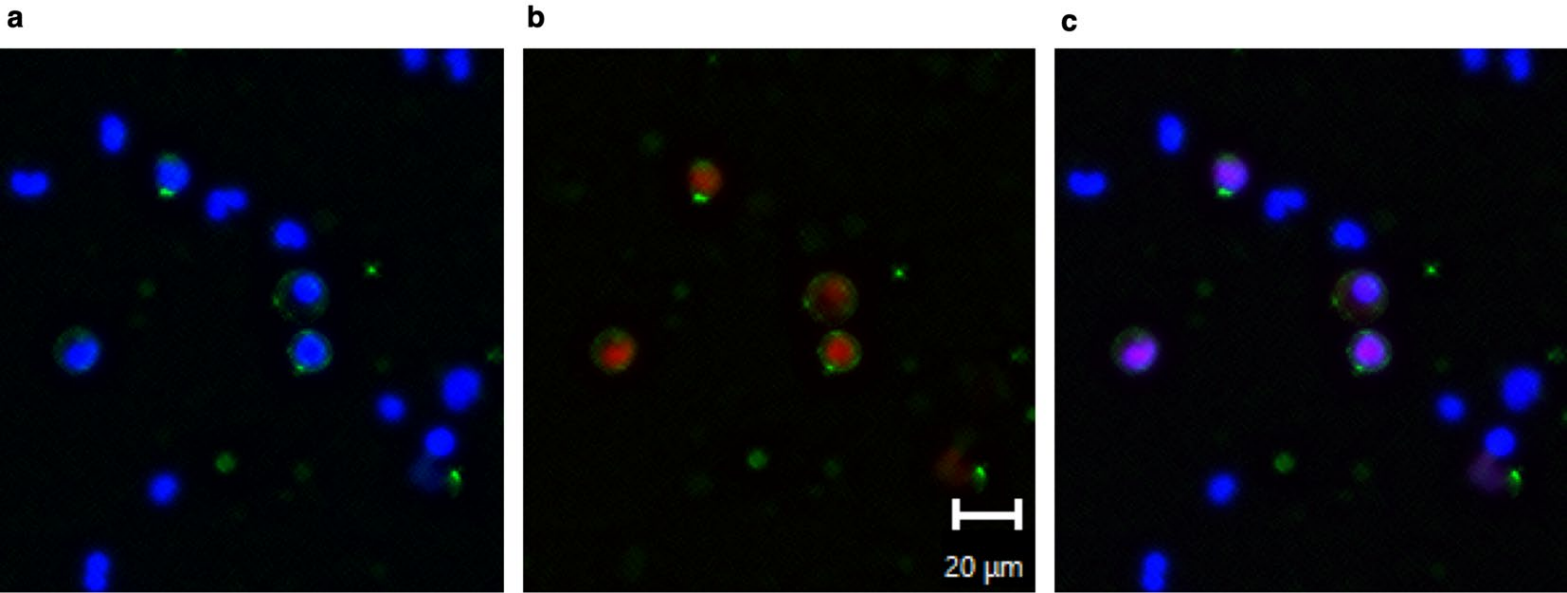
Representative apoptotic white blood cells in peripheral blood obtained from patients. (**a**) FITC-annexin V + Hoechst33342; (**b**) FITC-Annexin V + Eth D-III; (**c**) merged image. White blood cells are stained with FITC-annexin V (apoptosis: green on membrane), Hoechst33342 (all cells: blue on nucleus), and Eth D-III (necrotic cells: red on nucleus). Cells in figure A stained with green and blue represent primary apoptosis. Cells in figure C stained with green and purple represent primary necrosis.

The number of white blood cells (WBCs), neutrophils, lymphocytes and monocytes decreased significantly with HD (Table 1). The early apoptotic ratio of WBCs, neutrophils and monocytes increased significantly after HD, but no significant difference was observed for lymphocytes (Table 2). With regard to the type of dialyzer membrane, no differences were observed in numbers of neutrophils, or the increase in early apoptotic ratio between patients with polysulfone and polymethylmethacrylate (PMMA) membranes. Representative figures for apoptosis-positive cells are shown in Fig. 1.

**Table 1:** Changes in white blood cell count, neutrophils, lymphocytes, and monocytes.

	WBC	Neutrophile	Lymphocyte	Monocyte
Pre HD (/µL)	6,142 ± 2,474	4,467 ± 2,151	1,002 ± 513	398 ± 147
Post HD (/µL)	5,586 ± 2,560	4,055 ± 2,186	1,122 ± 687	373 ± 173
Corrected post HD (/µL)	5,365 ± 2,540	3,900 ± 2,171	895 ± 451	362 ± 194
p value (Pre HD—Post HD)	< 0.001	< 0.001	0.052	0.091
p value (Pre HD—Corrected Post HD)	< 0.001	< 0.001	0.002	0.043

WBC, white blood cell; (n = 70), Data were expressed as mean ± SD.

Corrected post HD, post-HD level corrected by hemoconcentration rate.

**Table 2:** Changes in apoptotic ratio of respective leukocyte fractions.

	WBC	Neutrophile	Lymphocyte	Monocyte
Pre HD (%)	10.60 ± 6.74	13.27 ± 9.05	4.07 ± 2.82	19.42 ± 10.80
Post HD (%)	13.69 ± 9.52	17.38 ± 12.06	4.51 ± 2.59	23.59 ± 14.83
Corrected post HD (%)	13.24 ± 9.40	16.83 ± 11.89	4.28 ± 2.37	22.87 ± 14.61
p value (Pre HD—Post HD)	< 0.001	< 0.001	0.220	0.005
p value (Pre HD—Corrected Post HD)	0.004	0.003	0.538	0.019

WBC, white blood cell; (n = 70), Data were expressed as mean ± SD.

Corrected post HD, post-HD level corrected by hemoconcentration rate.

Significant increases were seen in myeloperoxidase (MPO) and pentraxin 3 (PTX3) levels by HD, while significant decrease was found in thiobarbituric acid-reactive substances (TBARS) (Table 3). No significant change was seen in H_2_O_2_ expression (data not shown) in respective fractions of WBCs (n = 70).

**Table 3:** Changes in serum myeloperoxidase (MPO), malondialdehyde (MDA), and pentraxicin-3 (PTX3).

	MPO (ng/mL)	MDS (µM)	PTX3 (pg/mL)
Pre HD	822 ± 718	1.041 ± 0.333	5,593 ± 2,996
Post HD	1,122 ± 687	0.989 ± 0.276	6,766 ± 3,323
Corrected post HD	1,062 ± 627	0.945 ± 0.275	6,531 ± 3,276
p value (Pre HD—Post HD)	< 0.001	0.097	< 0.001
p value (Pre HD—Corrected Post HD)	0.002	0.002	< 0.001

MPO, myeloperoxidase; MDA, malondialdehyde; PTX3, pentraxicin-3 (n = 70).

Data were expressed as mean ± SD.

Corrected post HD, post-HD level corrected by hemoconcentration rate.

**Figure 2:**
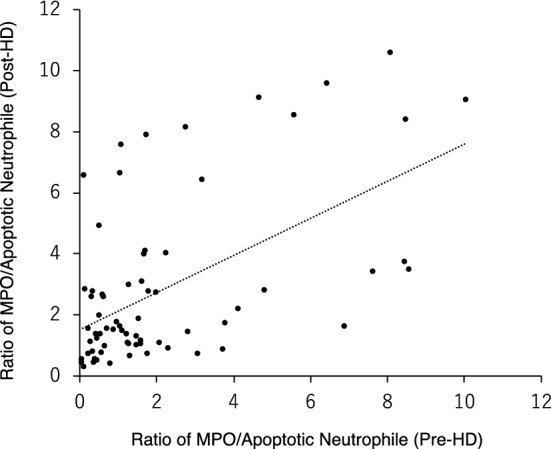
The relation between the pre and post HD levels of MPO/Apoptotic Neutrophiles ratio. A significant positive correlation exists between the two parameters (p < 0.001, r = 0.557). MPO: serum myeloperoxidase level (ng/mL). Apoptotic Neutrophiles: number of apoptotic neutrophiles (/µL).

Regarding the relationship between MPO levels and number of apoptotic neutrophiles, the ratios of (MPO level / number of apoptotic neutrophiles) in respective patients were 2.20 ± 2.47 at pre-HD and 2.85 ± 2.70 at post-HD, and there was a significant positive correlation between the two ratios (p < 0.001, r = 0.557; Fig. 2).

**Figure 3:**
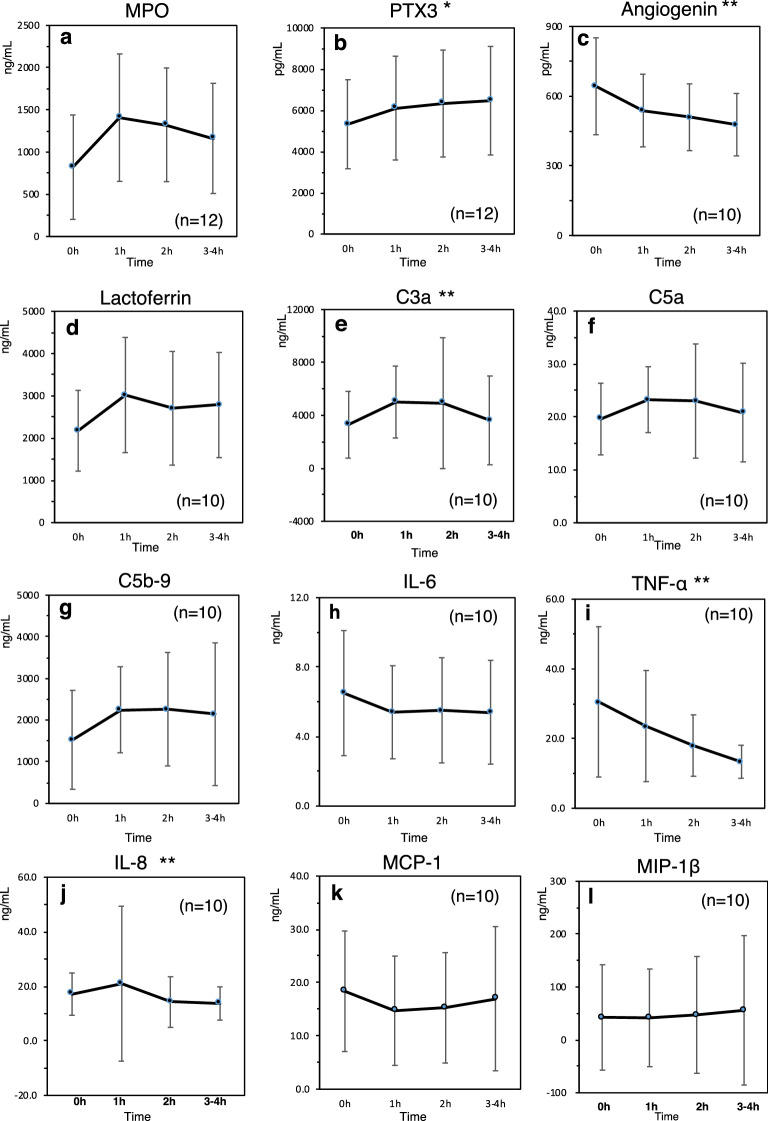
Time-course changes in myeloperoxidase (MPO) (**a**), pentraxicin-3 (PTX3) (**b**). angiogenin (**c**), lactoferrin (**d**), C3a (**e**), C5a (**f**), C5b-9 (**g**), IL-6 (**h**), TNF-α (**i**), IL8 (**j**), MCP-1 (**k**), and MIP-1β (**l**) during a 3–4 h hemodialysis session. (n = 10–12) *p < 0.01, **p < 0.05. Pre: Pre-HD level. Corrected post: post-HD level corrected by hemoconcentration rate. Data are expressed as mean ± SD.

In selected cases for whom blood samples were available during the course of HD (all on polysulfone membrane dialyzer), WBC counts and plasma parameters were measured (n = 10 to 12 for respective parameters). Changes of WBC fraction counts were as follows (n = 12); 6455 ± 857 (/μL) before HD, 5759 ± 1292 at 1 hr HD, 5365 ± 1275 at 2 h HD, and 5185 ± 752 after HD, respectively in WBCs (p = 0.01): 4823 ± 818 (/μL) before HD, 4374 ± 1288 at 1 hr HD, 4051 ± 1185 at 2 h HD, and 3848 ± 683 after HD, respectively in neutrophils (p < 0.01): 897 ± 348 (/μL) before HD, 818 ± 304 at 1 hr HD, 741 ± 268 at 2 h HD, and 793 ± 307 after HD, respectively in lymphocytes (not significant). Significant changes were seen in leukocyte counts, apoptosis ratio, MPO, angiogenin, and C3a (Fig. 3b,c,e). In terms of cytokine profiles, 12 of the 17 items were below the limit of detection, and data were obtained for only 5 items: IL6, IL8, monocyte chemotactic protein (MCP)-1 (MCP-1), macrophage inflammatory protein (MIP)- β (MIP-β), and TNF-α. TNF-α and IL-8 showed significant results (p = 0.032 and p = 0.011, respectively ) (Fig. 3i,j).

## Discussion

The quality of membrane materials and dialysis water had the great development in decades. It contributed the better biocompatibility of hemodialysis systems. However, mortality and morbidity rates are still unacceptably high among dialysis patients, It is possible that the various stress affect uremic patients and the pathophysiology of the actual biocompatibility status has not clearly elucidated in HD with advanced membrane material and ultrapure solution. The purpose of this study was to examine the impact of HD on peripheral leukocytes, with particular focus on neutrophils in patients using synthetic polymer membranes.

The main findings in this study can be summarized as follows: significant decrease in leukocyte counts by dialysis, significant increases in apoptosis-positive leukocytes by dialysis (neutrophils and monocytes), and significant increases in plasma MPO and PTX3 levels, with no or only marginal changes in plasma levels of pro-inflammatory cytokines and complement by dialysis.

Regarding the changes in leukocytes counts by dialysis, results from previous studies have not been constant. One study reported rapid decreases in WBCs 15–30 min after starting dialysis, followed by recovery to pre-dialysis levels after 1 h, resulting in an increase by the end of dialysis using polysulfone membrane^14^. Other reports have found that WBC count decreased after dialysis with cellulose acetate dialyzers^15^. Our results also showed a decrease in WBCs towards the end of dialysis. In addition, our results may imply that the susceptibility of leukocytes was more prominent for neutrophils. These findings may indicate that leukocytes may change distributions in the body after starting HD. Transient leukopenia has been confirmed to be caused by activation of the alternative pathway and lectin pathway, which are stimulated by the dialysis membrane^16^. In the present study, transient but significant increases were seen in the products of complement degradation by dialysis (plasma C3a), indicating that complement activation was not suppressed completely in those cases using a polymer membrane, as reported elsewhere^17^.

Interestingly, significant increases were seen in apoptosis-positive cells among neutrophils and monocytes, but not among lymphocytes, indicating that the susceptibility to induction of apoptosis during HD differs by cell type.

Regarding the increase in apoptotic cells, several mechanisms can be speculated. In terms of physiological processes, angiogenin in blood inhibits the degranulation of neutrophils^18^. In this study, significant decreases in plasma angiogenin were seen after dialysis, while significant increases were found in plasma MPO. Since MPO molecules present in azurophil or special granules in neutrophils, the decrease in angiogenin by dialysis would thus induce a degranulation reaction in neutrophils, resulting in increased apoptotic cells. The observations that increased ratio of apoptotic neutrophils by HD, and that a significant positive correlations between the pre and post HD levels in the ratio of (MPO/number of apoptotic neutrophiles), may well support the causal relationship between neutrophil apoptosis and MPO degranulation from neutrophile.

Other possible mechanisms are the activation of complement, which could induce neutrophil respiratory burst^19^, and direct injury through microcirculation stress from mechanical stimuli such as dialysis membranes, dialysis circuits and turbulence^20^. As for the latter possibility, we suppose that neutrophils mechanically stimulated on the dialyzer surface may undergo apoptosis, or alternatively, primed neutrophils present before HD may undergo apoptosis due to injury on the dialyzer surface. Changes in MPO during HD showed a unique pattern as reported elsewhere, with an initial rapid increase early after the dialysis session, markedly different from changes in lactoferrin which is another molecule present in plasma by neutrophil degranulation. And there were cases in whom changes of MPO levels by HD were significantly high, irrespective of the changes of apoptosis rate by HD. Taken together the observations, these data may indicate the unphysiological release of MPO from neutrophils into blood during dialysis, which may be involved in the mechanical burst of neutrophils.

Microinflammation in dialysis patients is a critical issue in terms of patient prognosis, but the exact mechanisms underlying this pathology have not been clearly elucidated. Stimulation of pro-inflammatory cytokines from mononuclear cells by dialysis comprises the classical hypothesis of the bio-incompatibility of HD. In the present study, among the various types of cytokines measured, no increases were seen in the plasma pro-inflammatory cytokines by dialysis, as recently reported elsewhere^8^. Thus, the pathological role of cytokines may be suppressed/lessened in the current dialysis therapy. However, in the present study, plasma PTX3, as a marker of inflammation, showed a gradual increase over time, suggesting that inflammation may have increased during dialysis^21^. Taking into account the process by which MPO mediates production of oxygen radicals in the presence of H_2_O_2_, we suppose the MPO release through neutrophil apoptosis, in addition to complement activation, may play an important role in the development of micro-inflammation during HD. Nevertheless, it is reported that CRP and Il-6 take longer to rise after an inflammatory stimulus^11^ and, therefore, this study may have failed to measure a rise of such slower responding cytokines during the 3 to 4 h duration HD session. This issue remained to be addressed. Regarding TBARS, a representative oxidative stress marker, it was shown to decrease by HD in the present study. This may indicate the suppressed oxidative stress during HD, which seems conflict to our speculation mentioned above. TBARS is a reactive product of thiobarbituric acid and aldehydes (MDA). However, TBARS does not always reflect the oxidative stress status during HD, because MDA is a small molecule, and thus passes through the membrane during HD. Accordingly, it is difficult to give a concrete conclusion as to the changes of redox state during HD in light of changes of TBARS. We think this issue also needs further clarification.

Repeated neutrophil damage during every dialysis session may be involved in increased susceptibility to infection, and MPO release enhances oxidative stress, resulting in a state of chronic microinflammation for dialysis patients^22^. Plasma MPO has been reported as an independent risk factor for all-cause mortality in HD patients^23,24^. In a previous report by Kamyar et al., MPO was higher in dialysis patients compared to non-dialysis controls, and cases with a particularly high MPO level showed a high death ratio of 1.81 compared to the moderate group^25^. Accordingly, we think that amelioration of neutrophil injury during HD may be clinically very important, in order to suppress MPO release into blood. This intervention may ameliorate microinflammation in HD patients and contribute to better prognosis. We have previously reported that dialysis water, which contains molecular hydrogen, could ameliorate neutrophil injury ex vivo, and application of this water to HD therapy could suppress high MPO levels^26^. Furthermore, clinical outcomes could also be improved. Other therapeutic interventions may be effective in ameliorating MPO levels through manipulating neutrophil injury during HD, such as on-line HDF, flow rate of blood volume, dialysate Ca ion level^27^, or use of a vitamin E-coating membrane^28^. These issues need to be elucidated in future.

Several limitations of the present study should be noted. Firstly, in patient selection, there are many cases of complications, including cancer, and malnutrition. Secondly, there are no on-line HDF cases registered. Third, the mechanism by which dialysis increases apoptosis has not been elucidated. Forth, the CD marker that evaluates degranulation was not measured, and this may reduce data accuracy of the present study. Fifth, leukocyte adhesion and deposition on the membrane and bubble trap filter could not be evaluated correctly. And lastly, the clinical impact of dialysis-induced leukopenia and apoptosis is unknown, we think that it needs to be clarified, especially in the light of immune dysfunction, and susceptibility to infection.

In summary, the bio-incompatibility of HD has remained a crucial issue, even with current systems. Among several pathologies, the process of neutrophil apoptosis accompanying MPO degranulation may be involved with the development of microinflammation. To achieve better patient outcomes, innovative HD systems that fully improve biocompatibility are needed.

## Methods

### Patients

Patients comprised 70 patients on HD who were treated at Tohoku University Hospital (Miyagi, Japan) between June 2017 and May 2019. All patients had been receiving HD regularly 3 times a week for 3–4 h/session, using a high-performance bio-compatible membrane (polysulfone [n = 54], including vitamin E-bonded type [n = 35] or poly-ester type [n = 1]; or PMMA [n = 16]). Ultra-pure water with no detectable endotoxin was used for dialysate manufacturing. Patient characteristics are shown in Table 4 (51 males, 19 females; mean age, 63 ± 14.5 years; median duration of HD, 18 months). The leading cause of underlying kidney disease was diabetic kidney disease (40%), and 46% of patients had a history of CVD. Informed consent was obtained from all participants, and the study protocol was fully approved by the ethics committee at Tohoku Medical University (approval number 2017-1-134). All methods were carried out in accordance with relevant guidelines and regulations.

**Table 4:** Patients demographics.

n	70
Age (years old)	63 ± 15.4
Male (%)	51 (73)
Dialysis vintage (months)	18 (1–391)
Pre-HD body weight (kg)	60.3 ± 14.5
BMI (kg/m^2^)	23.1 ± 4.8
**Underlying kidney disease (%)**	
Diabetic kidney disease	28 (40)
Nephrosclerosis	12(17)
Glomerulonephritis	13(19)
Others	17 (24)
Comorbidities (%)	
CVD	28 (40)
Current cancer	18 (26)
Apoplexy	13 (19)
**Dialysis period (%)**	
Initiation (< 3 months)	25 (36)
Maintenance	45 (64)
**Dialysis membrane**	
PMMA (%)	16 (23)
PS (%)	54 (77)
**Laboratory data (Pre-HD)**	
WBC (/μL)	6142 ± 2474
Hemoglobin (g/dL)	9.9 ± 1.3
Platelet (× 10^4^/μL)	19.2 ± 8.1
Total protein (g/dL)	6.2 ± 0.8
Albumin (g/dL)	2.8 ± 0.6
BUN (mg/dL)	56 ± 25
Creatinine (mg/dL)	8.91 ± 2.89
Na (mEq/L)	136 ± 4
K (mEq/L)	4.1 ± 0.6
Cl (mEq/L)	104 ± 4
Calcium (mg/dL)	8.5 ± 0.8
Phosphate (mg/dL)	5.2 ± 1.6
CRP (mg/dL)	2.11 ± 4.71
β2- microglobulin (mg/L)	24.8 ± 7.5

Values are given as mean ± SD or median (minimum to maximum).

HD; hemodialysis, BMI; body mass index, CVD; cardiovascular disease.

PMMA; polymethylmethacrylate, PS; polysulfone, WBC; white blood cell.

BUN; blood urea nitrogen, CRP; C-reactive protein.

## Methods

Blood samples were obtained from all patients before and after the HD session (two samples each), for collection of plasma and whole blood cells. One of the blood samples was immediately centrifuged with ethylenediamine tetra-acetic acid, and the plasma was stored at − 80 °C until needed for measurements. WBCs were separated from the second blood sample by hemolyzing red blood cell with Lysing Buffer (Becton, Dickinson and Co., Franklin Lakes, NJ) within 1.5 h after sampling. The WBC sample was then separated into two samples to measure apoptosis and necrosis, and hydrogen peroxide (H_2_O_2_). For the measurements of apoptosis and necrosis, a 100-µL sample containing WBCs was stained with 5 µL each of reagent (annexin-V and propidium iodide (PI**)**; FITC Annexin V Apoptosis Detection Kit I; Becton, Dickinson and Co.) in the dark for 15 min. Thereafter, 100,000 cells were measured with the blue laser (488 nm) of a flow cytometer (SH800 Cell Sorter; Sony, Tokyo, Japan). Further, H_2_O_2_ staining (BES-H2O2; Wako Chemical Inc., Tokyo, JP) was performed simultaneously. The sample was reacted with 5 µL of reagent in the dark for 15 min, and 10,000 cells were measured with the blue laser of the flow cytometer. In 3 cases, WBCs suspension was subjected to examine with another kit (Apoptotic/Necrotic/Healthy Cells Detection Kit; PromoCell, Heidelberg, DE; FITC-Annexin V in TE buffer containing 0.1% NaN3, Ethidium Homodimer(EthD) -III (EthD -III), Hoechst 33342 in PBS) to stain apoptotic, necrotic, and all cells and confirmed by fluorescence microscope (BZ-8100; Keyence, Osaka, JP).

Preserved plasma samples underwent measurement of the following parameters: total protein (TP) (Unicel DxC800; Beckman coulter, Brea, US), MPO (Human Myeloperoxidase ELISA kit; Abcam plc., Cambridge, UK), TBARS as a marker of malondialdehyde (MDA) by measurement reported elsewhere^13^, PTX3 (Human Pentraxin 3 ELISA; BioVendor, Brno, CZ), angiogenin (Human Angiogenin Quantikine ELISA Kit; R&D Systems, Minneapolis, MN), lactoferrin (Human Lactoferrin ELISA Kit, Abcam plc.), complement components C3a, C5a, and C5b-9 (Micro Vue complement C3a Plus ELIA, C5a Plus ELISA, and SC5b-9 Plus ELIA; QUIDEL, San Diego, CA). Furthermore, measurement of cytokines IL-1, IL-2, IL-4, IL-5, IL-6, IL-7, IL-8, IL-10, IL-12, IL-13, IL-17, granulocyte colony-stimulating factor (G-CSF), granulocyte macrophage colony-stimulating factor (GM-CSF), interferon (IFN)-γ, MCP-1, MIP-β, and TNF-α (Bio-Plex Pro Human Cytokine Assays 17-plex; Bio-Rad Laboratories, Hercules, CA) were performed on preserved plasma using ELISA kits.

Fresh dialysate was subjected to measure the level of endotoxin once a month regularly by using special device (Toxinometer ET-Mini wired Set, Wako, Osaka, JP). During the period of study, the endotoxin concentration in the dialysate was below the measurement limit value (< 0.7 EU/L).

In the present study, we considered the hemoconcentration effect by HD when comparing measured values of leukocytes and molecules between levels of pre and post HD. We calculated hemoconcentration rate by using TP levels (g/dL) as follows: Rate (%) = 100 × (Post-HD TP level – Pre-HD TP level) / Pre-HD TP level). Then the corrected post-HD levels were determined using the following formula: Corrected post HD levels = measured post-HD levels × (1-Rate/100)). We corrected the data of time course changes in a similar way (Fig. 3).

### Analysis

Variables were expressed as mean ± standard deviation (SD) or median (minimum to maximum), as appropriate. Statistical significance was set at the level of P < 0.05.

Statistical analyses for comparison and correlation were performed using paired t-test, non-parametric Wilcoxon paired rank test, and Pearson's correlation test. Time courses were analyzed using multivariate analysis of variance (MANOVA) or repeated-measures analysis of variance, and Bonferroni for multiple comparison procedure. All statistical analyses were performed with JMP Pro version 15.0 (SAS Institute Inc., Cary, NC).

## References

[1] de Jager DJ (2009). Cardiovascular and noncardiovascular mortality among patients starting dialysis. JAMA.

[2] Steiber AL, Handu DJ, Cataline DR, Deighton TR, Weatherspoon LJ (2003). The impact of nutrition intervention on a reliable morbidity and mortality indicator: the hemodialysis-prognostic nutrition index. J. Ren. Nutr..

[3] El-Gamal D (2014). The urea decomposition product cyanate promotes endothelial dysfunction. Kedney Int..

[4] Hakim RM (2000). Clinical implications of biocompatibility in blood purification membranes. Nephrol. Dial. Transplant..

[5] Bommer J, Jaber BL (2006). Ultrapure dialysate: facts and myths. Semin Dial..

[6] Carracedo J, Ramírez R, Martin-Malo A, Rodríguez M, Aljama P (2002). The effect of LPS, uraemia, and haemodialysis membrane exposure on CD14 expression in mononuclear cells and its relation to apoptosis. Nephrol. Dial. Transplant..

[7] Canaud B (1999). Imbalance of oxidants and antioxidants in haemodialysis patients. Blood Purif..

[8] Tsuruoka S, Sugimoto K, Hayasaka T, Fujimura A (2000). Ranitidine clearance during hemodialysis with high-flux membrane: comparison of polysulfone and cellulose acetate hemodialyzers. Eur. J. Clin. Pharmacol..

[9] Namayama M (2018). Novel haemodialysis (HD) treatment employing molecular hydrogen (H2)-enriched dialysis solution improves prognosis of chronic dialysis patients: a prospective observational study. Sci. Rep..

[10] Bouman CS, van Olden RW, Stoutenbeek CP (1998). Cytokine filtration and adsorption during pre- and postdilution hemofiltration in four different membranes. Blood Purif..

[11] Andreoli MCC (2007). Impact of dialyzer membrane on apoptosis and function of polymorphonuclear cells and cytokine synthesis by peripheral blood mononuclear cells in hemodialysis patients. Artif. Organs..

[12] Assa S (2014). Hemodialysis-induced regional left ventricular systolic dysfunction and inflammation: a cross-sectional study. Am. J. Kidney Dis..

[13] Friedrich B (2006). Acute effects of hemodialysis on cytokine transcription profiles: evidence for C-reactive protein-dependency of mediator induction. Kidney Int..

[14] Tien M, Aust SD (1982). Rabbit liver microsomal lipid peroxidation the effect of lipid on the rate of peroxidation. Biochim. Biophys. Acta..

[15] Craddock PR, Fehr J, Dalmasso AP, Brighan KL, Jacob HS (1977). Hemodialysis leucopenia. Pulmonary vascular leukostasis resulting from complement activation by dialyzer cellophane membranes. J. Clin. Invest..

[16] Yoon JW, Pahl MV, Vaziri ND (2007). Spontaneous leukocyte activation and oxygen-free radical generation in end-stage renal disease. Kidney Int..

[17] Inoshita H (2012). An analysis of functional activity via the three complement pathways during hemodialysis sessions: a new insight into the association between the lectin pathway and C5 activation. Clin. Kidney J..

[18] Yamamoto T (2013). Changes in circulating biomarkers during a single hemodialysis session. Hemodialysis Int..

[19] Schmaldienst S, Oberpichler A, Tschesche H, Hörl WH (2003). Angiogenin: a novel inhibitor of neutrophil lactoferrin release during extracorporeal circulation. Kidney Blood Press. Res..

[20] Ehlenberger AG, Nussenzweig V (1977). The role of membrane receptors for C3b and C3d in phagocytosis. J. Exp. Med..

[21] Asadinezhad A, Lehocký M, Sáha P, Mozetič M (2012). Recent progress in surface modification of polyvinyl chloride. Materials (Basel)..

[22] Argani H (2012). Serum Fetuin-A and Pentraxin3 in hemodialysis and renal transplant patients. Clin. Biochem..

[23] Nakayama M (2017). Oral ferric citrate hydrate associated with less oxidative stress than intravenous saccharated ferric oxide. Kidney Int. Rep..

[24] Kim JK (2020). Prognostic role of circulating neutrophil extracellular traps levels for long-term mortality in new end-stage renal disease patients. Clin. Immunol..

[25] Nelson K (2011). Upregulation of surrogate markers of inflammation and thrombogenesis in patients with ESRD: pathophysiologic and therapeutic implications. Clin. Appl. Thromb. Hemost..

[26] Kalantar-Zadeh K, Brennan ML, Hazen SL (2006). Serum myeloperoxidase and mortality in maintenance hemodialysis patients. Am. J. Kidney Dis..

[27] Nakayama M (2010). A novel bioactive haemodialysis system using dissolved dihydrogen (H_2_) produced by water electrolysis: a clinical trial. Nephrol. Dail. Transplant..

[28] Höri WH, Haag-Weber M, Mai B, Massry SG (1995). Verapamil reverses abnormal [Ca2+]i and carbohydrate metabolism of PMNL of dialysis patients. Kidney Int..

[29] Senatore M (2002). Immune dysfunction and cytokine production in hemodialysis. Nephron..

